# Age- and sex-specific prevalence of chronic comorbidity in adult patients with asthma: A real-life study

**DOI:** 10.1038/s41533-019-0127-9

**Published:** 2019-04-29

**Authors:** Mathijs Veenendaal, Janine A. M. Westerik, Lisette van den Bemt, Janwillem W. H. Kocks, Erik W. Bischoff, Tjard R. Schermer

**Affiliations:** 1 Department of Primary and Community Care, Radboud Institute for Health Sciences, Radboud University Medical Center, Nijmegen, The Netherlands; 2 Department of Inhalation Medicine, Observational Pragmatic Research Institute, Singapore, Singapore

**Keywords:** Diagnosis, Asthma

## Abstract

The presence of comorbidity can be associated with poorer asthma outcomes. Previous prevalence studies focused on a limited selection of comorbid conditions in asthma only. We aimed to determine age- and sex-specific prevalence estimates for the full range of chronic comorbid conditions in adult asthma patients by performing a retrospective cohort study based on 32,787 medical records of patients aged ≥16 years with asthma from 179 general practices in the Netherlands. Age- and sex-specific prevalence estimates of 76 chronic comorbidities and 14 disease categories based on International Classification of Primary Care codes were determined. Chronic comorbidity was present in 65.3% of male asthma patients and 72.8% of female asthma patients, with female patients having a higher mean (SD) of 2.0 (2.1) comorbidities compared to male patients (1.7 (2.0)). This mean increased to 5.0 (2.7) conditions in the 75+ age group. Most prevalent comorbidities were hypertension (20.1%), osteoarthritis (11.5%), eczema (11.5%) and dyspepsia (10.7%). Compared to male asthma patients, female asthma patients showed higher odds for the presence of other chronic conditions in eight disease categories. Neurological (odds ratio [OR]; 95% confidence interval 2.01; 1.76–2.29), blood forming/lymphatics (OR 1.83; 1.38–2.42) and musculoskeletal diseases (OR 1.82; 1.69–1.95) showed the highest association with female sex. In conclusion, the presence of chronic comorbidity is the norm in adults with asthma and it is more prevalent in female than in male asthma patients. The odds of having a specific comorbid condition may differ between the sexes. Attention in guidelines on how to handle comorbidities may lead to a more targeted treatment for comorbidities and more patient-centred asthma management.

## Introduction

Bronchial asthma is a common chronic pulmonary disease, which is characterized by inflammation, obstruction and hyperresponsiveness of the bronchial tree.^1^ In the majority of patients with asthma the disease is mild to moderate and treated in general practice. In the Netherlands 31.8 per 1000 men and 40.5 per 1000 women consult a general practitioner (GP) with asthma complaints every year.^2^

Asthma frequently co-exists with other diseases^3^ and the presence of this comorbidity can be associated with poorer asthma control, lower quality of life, higher morbidity and higher mortality.^4–7^ The identification and management of comorbidity is therefore increasingly recognized as an important part of asthma management.^8^

Several specific comorbidities like (allergic) rhinosinusitis, gastro-oesophageal reflux disease (GERD) and other respiratory diseases like chronic obstructive pulmonary disease (COPD) are known to be more prevalent in patients with asthma.^9^ Often these studies specifically included a hospital care population, thus representing patients in the more severe part of the asthma severity spectrum only.^10^ Other studies only focused on specific age groups like children or the elderly and are therefore not representative for the asthma population at large.^11,12^

Several previous studies have looked at the association between asthma and other chronic diseases in general population samples.^13–17^ Compared to non-asthma controls, asthma seems to be weakly associated with a higher prevalence of comorbidity in different disease categories like cardiovascular, other respiratory and mental diseases. However, these studies investigated only an a priori, limited selection of chronic conditions or major disease categories, or did not necessarily represent the asthma patient population that is managed in general practice. Therefore, comorbidity prevalence estimates from these studies may not reflect the true burden of comorbidity in the general practice asthma patient population. Therefore, the main aim of this study was to determine sex- and age-specific prevalence estimates for the *full range* of chronic comorbid conditions in adult asthma patients in real-life general practice. As our aim was not to look whether or not patients with asthma have an increased risk of other chronic conditions, we chose not to include a control group of non-asthma subjects in our study.

## Results

### Study population

Data from 40,050 patients aged ≥16 years with an asthma diagnosis were available. Of these patients, 7263 were excluded because they did not meet the criterion of having an ‘active’ diagnosis of asthma. Table 1 shows baseline characteristics of the final study population (*n* = 32,787). Just over 40% (40.7%, *n* = 13,348) were male patients. Mean (SD) age at the start of the study period (on 1 January 2012) was 46.3 (17.7) years. Eight percent (8.2%, *n* = 2704) of patients were lost to follow-up before the end of the 2-year observation period. Reason for loss to follow-up was known for 44.9% of patients, with moving out of the practice being the predominant reason. Patients lost to follow-up were 38.3% male patients, had a mean age of 45.2 (standard deviation (SD) 19.0) years and were comparable with the patients with complete follow-up (*n* = 30,083; 40.9% male, mean age 46.5 (17.5) years). Twelve percent (12.1%, *n* = 3964) of patients entered the cohort after the start of the study period.

**Table 1:** Characteristics of the study population (*n* = 32,787)

Patient characteristics	
Sex, male, *n* (%)	13,348 (40.7)
Age at 01-01-2012 (years); mean (SD; range)	46.3 (17.7; 16–110)
Age group, years, *n* (%)	
16–29	6824 (20.8)
30–44	8259 (25.2)
45–59	9514 (29.0)
60–74	6110 (18.6)
75+	2080 (6.3)
Lost to follow-up before 1-1-2014, *n* (%)	2704 (8.2)
Deceased, *n* (% of lost to follow-up)	166 (6.1)
Moved, *n* (% of lost to follow-up)	644 (23.8)
Nursing home, *n* (% of lost to follow-up)	9 (0.3)
General practice stopped providing data, *n* (% of lost to follow-up)	396 (14.6)
Unknown, *n* (% of lost to follow-up)	1489 (55.1)
Entering cohort after 1-1-2012, *n* (%)	3964 (12.1)
Use of inhaled corticosteroids 2012–2013^a^, *n* (%)	20,395 (62.2)

*SD* standard deviation, *ICS* inhaled corticosteroid

^a^At least one prescription ICS in the period 2012–2013

### Prevalence of chronic comorbidity

Table 2 shows the number of chronic comorbid conditions besides asthma, with an overall mean (SD) of 1.9 (2.1) conditions. The number of chronic comorbidities increased with age to a mean of 5.0 (2.1) comorbid conditions in patients aged 75 years and older. Generally, female asthma patients had a higher mean number of comorbid conditions compared to male asthma patients (*p* *<* 0.001), but this difference was no longer seen in the 75+ years age group.

**Table 2:** Mean number of chronic comorbid diseases, sorted by age group

Age (years)	Men (SD; range)	Women (SD; range)	Total (SD; range)	*P* value*
16–29	0.52 (0.74; 0–5)	0.72 (0.91; 0–7)	0.63 (0.85; 0–7)	<0.001
30–44	0.86 (1.15; 0–8)	1.18 (1.31; 0–8)	1.05 (1.26; 0–8)	<0.001
45–59	1.72 (1.69; 0–11)	2.05 (1.82; 0–12	1.91 (1.77; 0–12)	<0.001
60–74	3.26 (2.24; 0–17)	3.49 (2.27; 0–14))	3.40 (2.26; 0–17)	0.023
75+	5.02 (2.66; 0–14)	5.01 (2.65; 0–18)	5.02 (2.66; 0–18)	0.974
Total	1.72 (2.04; 0–17)	2.03 (2.13; 0–18)	1.90 (2.10; 0–18)^a^	<0.001

*SD* standard deviation, *COPD* chronic obstructive pulmonary disease

*P value calculated for the difference in mean between men and women

^a^As a reference: in a previous study in patients with COPD (aged ≥40 years) using the same general practice database, we observed an average number of 3.0 chronic comorbid diseases^33^

Almost 35% (34.7%) of male patients and 27.2% of female patients had only asthma. For all ages combined, 40.4% of male patients and 47.9% of female patients had two or more chronic conditions next to their asthma (*p* *<* 0.001).

Table 3 shows the prevalence estimates of the conditions with a total prevalence of >1.5% with the calculated odds ratios (OR) for female sex, adjusted for age. Hypertension (20.1%), osteoarthritis (11.5%), eczema (11.5%), dyspepsia (10.7%) and COPD (9.5%) were the most prevalent comorbidities. The conditions with the largest difference in prevalence between female and male patients were recurrent urinary tract infection (OR; 95% confidence interval [CI]: 9.27; 6.75–12.75), thyroid disorders (OR 5.03; 4.35–5.83), migraine (OR 4.35; 3.51–5.40) and osteoporosis (OR 4.19; 3.50–5.02), all to the disadvantage of women. Supplementary Appendices 1 and 3 provide the complete list of 76 comorbid conditions and their prevalence estimates, overall and by sex.

**Table 3:** Prevalence estimates of chronic comorbid diseases with a total prevalence >1.5%, sorted by total prevalence

Comorbidity	Total prevalence (%)	Prevalence in males (%)	Prevalence in females (%)	Odds ratio female sex (95% CI)
Hypertension	20.1	18.0	21.6	**1.26 (1.18–1.34)**
Osteoarthritis	11.5	8.4	13.7	**1.80 (1.66–1.95)**
Eczema	11.5	10.7	12.1	**1.17 (1.09–1.25)**
Dyspepsia, gastro-oesophageal reflux	10.7	10.1	11.2	**1.10 (1.02–1.18)**
COPD	9.5	10.7	8.6	**0.72 (0.67–0.78)**
Diabetes	8.5	8.6	8.4	0.92 (0.85–1.00)
Dyslipidemia	6.9	6.9	6.8	0.93 (0.85–1.02)
Chronic sinusitis	6.6	4.7	7.9	**1.73 (1.57–1.90)**
Obesity	6.5	4.4	8.0	**1.88 (1.70–2.07)**
Coronary heart disease	6.5	8.3	5.3	**0.52 (0.48–0.58)**
Blindness and low vision	6.1	5.6	6.4	1.05 (0.94–1.16)
Peripheral vascular disease	5.5	5.5	5.6	0.96 (0.87–1.06)
Irritable bowel syndrome	5.4	2.7	7.3	**2.84 (2.53–3.20)**
Thyroid disorder	5.1	1.6	7.4	**5.00 (4.32–5.80)**
Anxiety	4.9	3.2	6.0	**1.96 (1.75–2.19)**
Depression	4.6	2.9	5.7	**2.05 (1.83–2.31)**
Chronic kidney disease	4.4	3.6	4.9	**1.30 (1.16–1.47)**
Hearing loss	4.0	4.6	3.6	**0.67 (0.60–0.75)**
Stroke and transient ischaemic attack	3.4	3.5	3.3	**0.87 (0.77–0.99)**
Psoriasis	3.4	3.4	3.3	0.95 (0.84–1.08)
Skin cancer	3.0	2.9	3.0	0.98 (0.86–1.12)
Osteoporosis/osteopenia	2.9	1.0	4.2	**4.14 (3.44–4.98)**
Atrial fibrillation	2.9	3.3	2.6	**0.68 (0.59–0.78)**
Rheumatoid arthritis, other inflammatory polyarthritis and systemic connective tissue disorders	2.2	1.6	2.6	**1.64 (1.40–1.93)**
Diverticular disease of intestine	2.2	1.9	2.3	1.15 (0.98**–**1.34)
Migraine	2.1	0.7	3.1	**4.39 (3.54–5.44)**
Prostate disorders	2.0	4.9	–	–
Personality disorder	1.9	1.6	2.1	**1.40 (1.18–1.65)**
Heart failure	1.8	1.8	1.8	0.85 (0.72–1.02)
Recurrent urinary tract infection	1.8	0.3	2.8	**9.13 (6.64–12.55)**
Other chronic skin disease/neoplasm	1.7	1.6	1.8	1.16 (0.97–1.37)
Heart valve disease	1.7	1.8	1.7	0.86 (0.72–1.02)
Alcohol problems	1.7	2.6	1.0	**0.38 (0.32–0.45)**
Bronchiectasis/chronic bronchitis	1.6	1.5	1.6	1.08 (0.90–1.29)
Breast cancer	1.6	0.0	2.6	–

*CI* confidence interval, *COPD* chronic obstructive pulmonary disease

Odds ratios were adjusted for age. Odds ratios marked bold were statistically significant

### Prevalence of disease categories

**Fig. 1:**
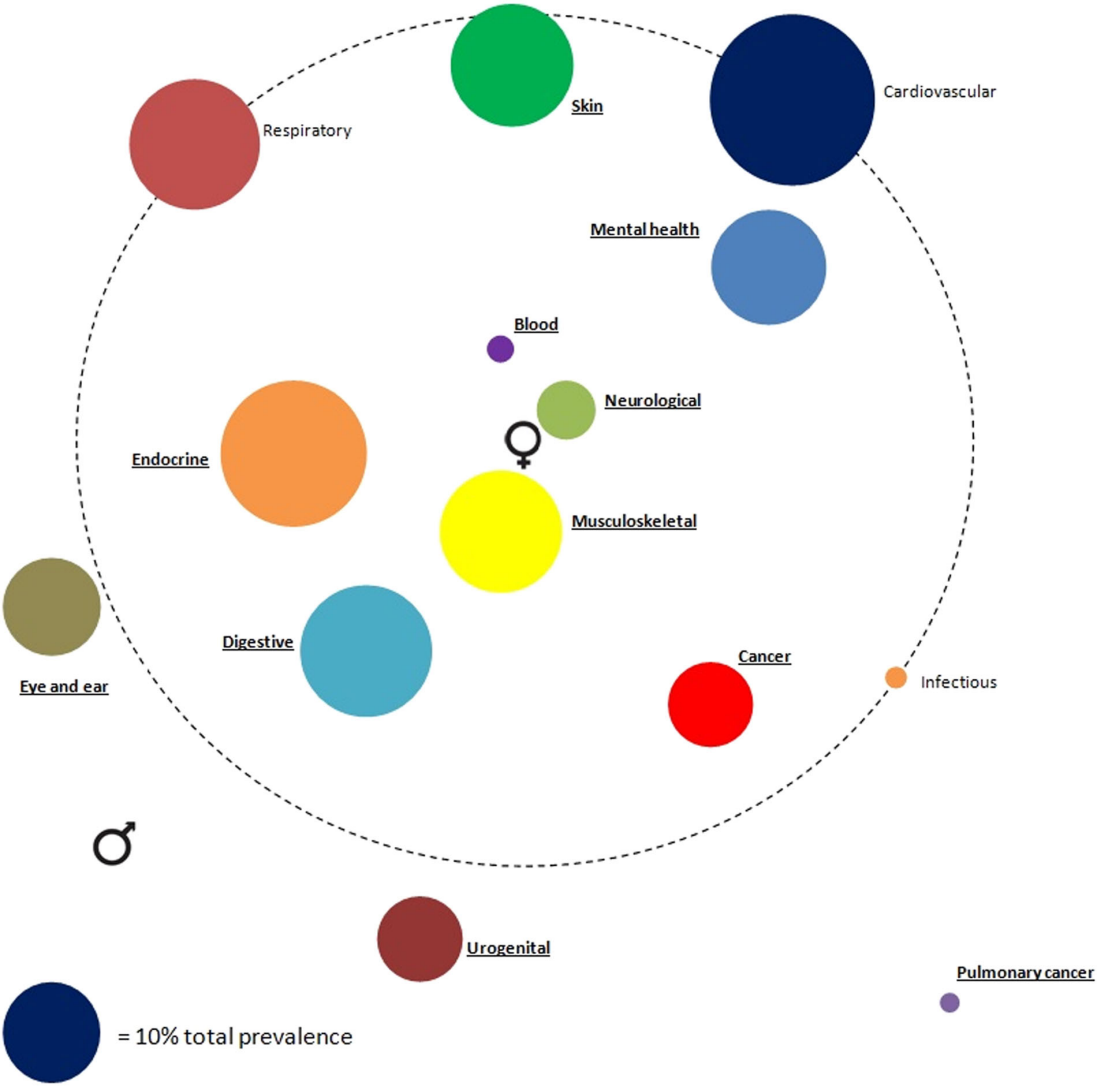
Comorbidome of disease categories in relation to sex. The diameter of each circle represents the total prevalence of the disease category in the study population, the distance to the centre of the circle represents the odds ratio for female sex, adjusted for age using logistic regression. Proximity to the centre represents a higher odds ratio for female sex. The large dashed circle represents an odds ratio of 1, and diseases outside the dashed circle are associated with male sex (odds ratio <1). Underlined disease categories had a statistically significant difference between men and women (OR > 1 or OR < 1). OR odds ratio

Figure 1 shows the prevalence estimates of 14 disease categories and their association with sex. Cardiovascular (28.7%) followed by endocrine (22.5%) and digestive (18.3%) diseases were most prevalent. Compared to male asthma patients, female asthma patients showed statistically significant higher odds for the presence of chronic conditions in eight disease categories. Neurological (OR; 95% CI: 2.01; 1.76–2.29), blood forming/lymphatics (OR 1.83; 1.38–2.42) and musculoskeletal diseases (OR 1.82; 1.69–1.95) showed the highest association with female sex. Female patients had lower odds of having pulmonary cancer (OR 0.59; 0.42–0.84), urogenital diseases (OR 0.82; 0.75–0.89) and eye/ear diseases (OR 0.89; 0.82–0.97).

**Fig. 2:**
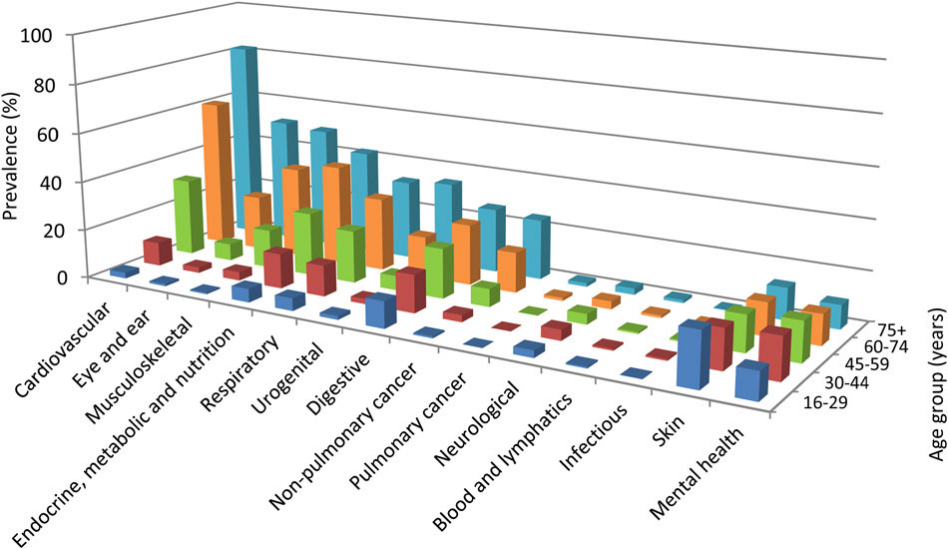
Prevalence of disease categories in the different age groups. The height of each bar represents the proportion (%) of patients in the respective age group suffering from at least one disease in the disease category

Figure 2 shows the age distribution of the 14 disease categories. Prevalence of most but not all disease categories increased with age, with cardiovascular diseases being most prevalent in the 75+ age group (82.1%), followed by eye/ear (51.2%) and musculoskeletal diseases (49.7%). Prevalence of skin diseases was highest in the 16–29 year age group (21.5%) and decreased with age. Mental health conditions had their peak at 30–44 years (16.7%), afterwards the prevalence decreased.

## Discussion

In this study, we determined the prevalence of chronic comorbid conditions in adult patients with asthma in general practice and described differences between sex and age groups within the asthma patient population. We observed that chronic comorbidity is the rule rather than the exception in adult patients with asthma, as 70% had at least one chronic comorbid condition next to their asthma. The mean number of chronic conditions was almost 2 and increased to a mean of 5 conditions in the 75+ age group. Cardiovascular, endocrine and digestive diseases were most prevalent. Female patients had more chronic comorbid conditions than male patients, with neurological, blood forming/lymphatics and musculoskeletal diseases showing the strongest association with female sex.

Our study confirms previous reports that chronic comorbidity is highly prevalent in patients with asthma.^13–16^ A population-based study in Scotland found that 62.6% of patients with asthma had at least one comorbid condition and that 16.3% had 4 or more.^16^ The higher proportion of patients with comorbidity observed in our study can be explained by the more extensive list of chronic conditions included in our analysis (39 versus 76 conditions, respectively), thus representing the ‘real’ prevalence of chronic comorbidity in the adult asthma patient population. The estimates of the most prevalent comorbid conditions in our study were comparable to other population-based and cohort studies, with hypertension being the most prevalent comorbid condition.^13,16^ Hypertension has been associated with poorer asthma control in previous studies.^18^ Other prevalent comorbidities in our study like GERD, eczema, COPD and sinusitis are also known to be associated with poorer asthma outcome.^1,19,20^ Prevalence of mental conditions in patients with asthma varies exceedingly in previous studies.^16,21–24^ Our prevalence estimates for depression (4.6%) and anxiety (4.9%) were in the lower range of this spectrum. This is probably due to the additional criterion to express chronicity in our study (recurrence of the depression episode within 24 months). Therefore, the prevalence estimates of these mental conditions as well as the other ‘conditionally chronic’ conditions in our study represent chronic instead of episodic conditions and are thus more clinically relevant.

In our study, more women than men suffered from asthma, which is in agreement with earlier findings.^25^ Sex differences in asthma severity have been described before.^26^ Cazzola et al.^13^ found that being female slightly increased the association between cardiovascular diseases, diabetes mellitus, dyslipidaemia, osteoporosis, mental diseases and GERD and asthma. Of these diseases, in our study only osteoporosis and mental diseases were associated with female sex, whereas GERD was associated with male sex. Our observation that cancer was associated with female sex and urogenital diseases with male sex should be interpreted with caution because these categories contain sex-specific diseases (i.e. ovarian cancer and prostate disorders).

This study’s major strength is the inclusion of almost 33,000 patients with asthma. Exclusion criteria were minimal, so this cohort can be considered as representative for the general adult asthma population. Moreover, we looked at the total range of chronic comorbid conditions as well as at ‘conditionally’ chronic and recurrent conditions without making an a priori selection. This results in more precise and real-life estimates of the prevalence of chronic comorbidity in adult patients with asthma compared to previous prevalence studies. Our additional analyses based on age groups and sex completed the comprehensive overview of the prevalence estimates of chronic comorbidity in patients with asthma in general practice.

This study also has some limitations, most of which are inherent to its observational and retrospective design. Firstly, because of the lack of a control group, it was only possible to determine the prevalence estimates of comorbid conditions in patients with asthma. However, the aim of our study was to determine the age- and sex-specific prevalence estimates of the ‘full range’ of chronic comorbid conditions in patients with asthma, not to find out whether or not patients with asthma have a higher risk of developing other chronic conditions, as this has already been investigated in several population-based studies.^13,16^

Secondly, inclusion of subjects with a misdiagnosis of asthma could have occurred because data from lung function tests were not available in our database. For the same reason, we were not able to avoid asthma and COPD misclassification. In a recently published study, a positive predictive value of 86.4% was found for asthma diagnosis using an asthma code in a general practice database, and information of neither spirometry nor use of asthma medication did significantly improve accuracy.^27^ Regarding the prevalence estimates of the comorbid conditions in our study, underestimation may have occurred due to under-registration and under-diagnosis (i.e. patients with asthma who have another chronic condition might not consult their GP for it).^28^ In addition, some conditions (e.g. depression) are less clear cut in terms of diagnostic labels, and coding conventions are likely to have influenced our prevalence estimates for such conditions.

Thirdly, we analysed sex differences by calculating OR for 76 different comorbid diseases. With this large number of male–female comparisons, some are bound to be ‘statistically significant’ at the 95% CI level. Moreover, female patients with asthma are more likely to seek care^26^, which will probably result in more comorbidity diagnoses. Finally, we used a study design with a dynamic cohort to determine period prevalence estimates of the chronic conditions. The strength of this type of cohort is a large, unbiased study population with minimal exclusion. However, patients lost to follow-up were included in the analysis and may have caused underestimation of the prevalence estimates, because these patients could have developed comorbidity between the moment of loss to follow-up and the end of the study period. Especially for gout, chronic sinusitis and recurrent urinary tract infection, this could have led to an underestimation, because these three conditions had an extra criterion of at least three episodes in 2011, 2012 and 2013 in order to be considered as chronic (Supplementary Appendix 1).

Like in COPD, the presence of chronic comorbidity in asthma patients may be associated with poorer symptom control and overtreatment (e.g. the use of angiotensin-converting-enzyme inhibitors for treating hypertension in asthma patients may cause cough which can be incorrectly attributed to asthma). But also the other way around, as asthma can interfere with the management of other comorbidities. For example, the use of inhaled corticosteroids may have a negative impact on diabetes control.^29^ The results of this study can be used by asthma guideline authors to provide health care professionals with better insight in the prevalence of chronic comorbidity in adults with asthma in different age groups and how this varies between the sexes.

It would be interesting to study a possible influence of the presence of comorbid conditions on the use of asthma medication (e.g. if the presence of comorbidity increases or decreases the use of inhaled corticosteroids) and asthma outcomes (e.g. the risk of an asthma exacerbation). Regarding the presence of comorbidity in patients included in clinical trials, guideline and health policy makers should be careful in interpreting the generalizability of these trials, as there is a need for ‘real-life’ trials that include patients with comorbidity.^30^

In conclusion, this study showed that chronic comorbidity is highly prevalent in adult patients with asthma, even more in women than in men. It provides a comprehensive overview of the prevalence estimates of the full range of chronic comorbid conditions in patients with asthma. It is important that health care professionals who are involved in asthma care assess the role of comorbidity in a specific patient in order to achieve patient-centred asthma management.

## Methods

### Study design

We conducted a retrospective cohort study, using a general practice database from the Department of Primary and Community Care at the Radboud University Medical Centre, Nijmegen, The Netherlands. In the Netherlands the GP is the gatekeeper to secondary care. GPs are informed about any new diagnoses in their patients by the medical specialists they refer their patients to. Thus, a patient’s electronic medical record in general practice contains information regarding all of his diagnoses. De-identified electronic medical records of patients diagnosed with asthma from 179 general practices in the Eastern part of the Netherlands were available. For each registered subject, the following information was available: age, sex, all diagnoses coded according to the International Classification of Primary Care first edition (ICPC-1) extended with Dutch ICPC sub-codes (https://www.nhg.org/themas/publicaties/icpc-online), all contacts with the GP, and all medication prescriptions with start and end dates categorized using the Anatomical Therapeutic Chemical classification system.^31^

### Study population

Study subjects aged ≥16 years (the Dutch GP asthma treatment guidelines consider patients ≥16 years as adults^32^) with physician-diagnosed asthma (ICPC-1 code R96) were included if they were registered at any moment between 1 January 2012 and 31 December 2013 in one of the 179 general practices that provided data. To avoid inclusion of misdiagnosed patients (i.e. patients with a childhood diagnosis of asthma without confirmation of this diagnosis later in life), patients were excluded if they had not had at least one asthma-related contact with their GP or at least one prescription for asthma medication after 1 January 2011. The cohort was a dynamic cohort, that is, patients lost to follow-up or patients entering the cohort after 1 January 2012 were included in the analysis. Patients were classified in the following age groups, based on their age at 1 January 2012: 16–29, 30–44, 45–59, 60–74, and ≥75 years.

### Ethics approval and consent to participate

In the Netherlands, all patients are listed with a GP and have access to specialized health care through this GP. For this database study, approval of an ethics committee was not required.

### Chronic comorbidities

An extensive list of chronic comorbid conditions was compiled based on definitions of chronic comorbidity in existing literature.^2,33–36^ All chronic conditions were considered as comorbidities regardless whether the condition had been diagnosed before or after the diagnosis of asthma (until 31 December 2013). Chronic comorbid conditions that had already been diagnosed before 2012 were also included in the analysis. We also investigated several recurrent conditions and applied criteria to define their chronicity based on Dutch general practice guidelines.^32^ Examples of these ‘conditionally’ chronic comorbidities are depression, anxiety, dyspepsia, urinary tract infection and gout. Specifications of these criteria can be found in Supplementary Appendix 1. Eventually, a total of 76 comorbidities were studied. The following 14 disease categories were made after clustering the separate comorbidities based on existing literature^33,36^: cardiovascular, respiratory, endocrine, digestive, skin, musculoskeletal, mental health, eye/ear, urogenital, neurological, blood forming/lymphatics, infectious, non-pulmonary cancer and pulmonary cancer (Supplementary Appendix 2).

### Outcomes and statistical analysis

Period prevalence estimates with 95% CIs for the 76 chronic comorbid conditions were calculated for the period 1 January 2012 until 31 December 2013 by dividing the number of asthma patients registered with the comorbid condition by the final study population (i.e. patients that were part of the asthma cohort at any time during the study period). Subgroups based on sex were analysed separately and ORs adjusted for age were calculated to analyse differences in the prevalence of chronic conditions and disease categories between male and female patients with asthma using logistic regression. A simple count of the number of comorbid chronic conditions was analysed per sex and age group using Poisson regression. *χ*^2^ tests were performed for categorized variables. Statistical significance was defined as *p* < 0.05. All analyses were performed using IBM SPSS statistical software version 22.

### Reporting summary

Further information on research design is available in the Nature Research Reporting Summary linked to this article.

## Supplementary information

Appendices

Reporting Summary

## Data Availability

The data and codes are available for other researchers. A motivated request can be sent to the corresponding author.

## Supplementary information

**Supplementary information** accompanies the paper on the *npj Primary Care Respiratory Medicine* website (10.1038/s41533-019-0127-9).
