# Supplementary material for: Age- and sex-specific prevalence of chronic comorbidity in adult patients with asthma: A real-life study
Source: NPJ Prim Care Respir Med. 2019 Apr 29;29:14. doi: 10.1038/s41533-019-0127-9 (PMC6488608; doi:10.1038/s41533-019-0127-9)
Supplement: Supplementary file 1 — Appendices [file 41533_2019_127_MOESM1_ESM.pdf]

Appendix 1 Prevalence estimates with 95% confidence-interval of 76 chronic comorbid diseases, sorted by prevalence (%)

**Comorbidity**	**Diagnoses**	**Prevalence, % (95%-CI)**	**ICPC 1 codes**	**Additional criteria**
Hypertension	Hypertension	20.1 (19.7-20.5)	K86, K87	
Osteoarthritis	Osteoarthritis/spinal spondylosis	11.5 (11.2-11.8)	L84, L84.01, L84.02	
	Knee osteoarthritis		L90	
	Hip osteoarthritis		L89	
	Osteoarthritis, other		L91	
Eczema	Atopic dermatitis	11.5 (11.2-11.8)	S87	
Dyspepsia, Gastoesophageal reflux	Stomach ulcer	10.7 (10.4-11.0)	D86.01	Recode OR connection to episode 12 months after first ICPC
	Duodenal ulcer		D85	Recode OR connection to episode 12 months after first ICP
	Peptic ulcer, other		D86	Recode OR connection to episode 12 months after first ICPC
	Oesophagus reflux with and without oesophagitis		D87, D87.01, D87.02, D84.02, D84.03, D84 (Only D84.01, D84.05 not)	Recode OR connection to episode 12 months after first ICPC
COPD	COPD	9.5 (9.2-9.8)	R95	
Diabetes	DM1, DM2	8.5 (8.2-8.8)	T90, T90.01, T90.02	
Dyslipidemia	Hypercholesterolemia/ hypertriglyceridemia	6.9 (6.6-7.2)	T93, T93.01, T93.02, T93.03, T93.04	
Chronic sinusitis	Chronic sinusitis	6.6 (6.3-6.9)	R75.02	
	Acute Sinusitis		R75.01 and R75	Recode OR connection to episode ≥3x/year in 2011, 2012 or 2013. Year starts from First ICPC code. Period between new episodes should be at least 29 days.
Obesity	Obesity	6.5 (6.2-6.8)	T82	
Coronary heart disease	Myocardial infarction / other ischemic heart disease	6.5 (6.2-6.8)	K75, K76, K76.02, K76.01	
Blindness & low vision	(Diabetic/hypertensive) retinopathy	6.1 (5.8 -6.4)	F83, F83.01, F83.02	
	Macular degeneration		F84	
	Blindness / amblyopia		F94	
	Cataract		F92, F92.01	
Peripheral vascular disease	Atherosclerosis	5.5 (5.3-5.7)	K91	
	Intermittent claudication / Raynaud / Buerger		K92, K92.01, K92.02, K92.03	
	Other disease cardiovascular system		K99, K99.01, K99.02, K 99.03, K99.04, K99.05,99.06	
Irritable bowel syndrome	Irritable bowel syndrome	5.4 (5.2-5.6)	D93	
Thyroid disorder	Hypothyroidism	5.1 (4.9-5.3)	T86	
	Hyperthyroidism		T85	
Anxiety	Anxiety disorder	5.0 (4.7-5.2)	P74, P 74.01, P74.02	Recode OR connection to episode 24 months after first ICPC code
	(chronic) functional somatic symptoms		P01, P78	Recode OR connection to episode 24 months after first ICPC code
	Phobia		P79.01	Recode OR connection to episode 24 months after first ICPC code
	Obsessive - compulsive disorder		P79.02	Recode OR connection to episode 24 months after first ICPC code
	Post-traumatic stress disorder		P02.01(Not P02)	Recode OR connection to episode 24 months after first ICPC code
	Somatoform disorder		P75	Recode OR connection to episode 24 months after first ICPC code
Depression	Depressive disorder	4.6 (4.4-4.8)	P76, P76.01	Recode OR connection to episode 24 months after first ICPC code
Chronic kidney disease	Renal insufficiency	4.4 (4.2-4.6)	U99.01 (Not: U99.02, U99.03, U99.04, Include U99 if there is no subcode available)	
Hearing loss	Deafness	4.0 (3.8-4.2)	H84, H86, H85	
	Otosclerosis		H83	
Stroke&transient ischaemic attack	TIA (transient ischemic accident)	3.4 (3.2-3.6)	K89	
	CVA		K90, K90.01, K90.02, K90.03	

Appendix 1 Prevalence estimates with 95% confidence-interval of 76 chronic comorbid diseases, sorted by prevalence (%), continued

Psoriasis	Psoriasis	3.4 (3.2-3.6)	S91	
Skin cancer	Skin cancer	3.0 (2.8-3.2)	S77.01, S77.02, S77.03, S77.04, S77	
Osteoporosis/osteopenia	Osteoporosis / osteopenia	2.9 (2.7-3.1)	L95.02, L95, L95.01	
Atrial fibrillation	Atrial fibrillation / flutter	2.9 (2.7-3.2)	K78	
Rheumatoid arthritis, other inflammatory polyarthritis & systemic connective tissue disorders	Rheumatoid arthritis / ankylosing spondylarthritis	2.2 (2.0-2.4)	L88.01, L88.02, L88	
Diverticular disease of intestine	Colonic diverticula, diverticulitis	2.2 (2.0-2.4)	D92	
Migraine	Migraine	2.1 (1.9-2.3)	N89	Recode OR connection to episode 12 months after first ICPC
Prostate disorders	Prostatic hyperplasia / hypertrophy	2.0 (1.8-2.2)	Y85	
Personality disorder	Personality disorder	1.9 (1.8-2.0)	P80, P80.01, P80.02	
Heart failure	(congestive) heart failure<br>Pulmonary heart disease	1.8 (1.7-1.9)	K77, K77.01, K77.02<br>K82	
Recurrent urinary tract infection	Urinary tract infection, chronic / recurrent	1.8 (1.7-1.9)	U71, U71.01, U71.02	Recode OR connection to episode ≥3 times / year in 2011, 2012 or 2013. Year starts from First ICPC code. Period between new episodes should be at least 8 weeks.
Other chronic skin disease / neoplasm (sub)cutis	Neoplasm cutis, subcutis non-specified<br>Vitiligo / lichen planus	1.8 (1.7-1.9)	S80, S80.01, S81, S83, S83.01, S83.02<br>S99.04, S99.06 (S99 without subcodes not included)	
Heart valve disease	Heart valve disease<br>Heart valve disease (rheumatic)	1.7 (1.6-1.8)	K83, K83.01, K83.02<br>K71.02 (if only K71, not included)	
Alcohol problems	Chronic alcohol misuse	1.7 (1.6-1.8)	P15, P15.01, P15.02, P15.03, P15.04, P15.05, P15.06	
Bronchiectasis / chronic bronchitis	Bronchiectasis/ Chronic bronchitis	1.6 (1.5-1.7)	R91.02, R91, R91.01	
Breast cancer	Breast cancer	1.6 (1.5-1.7)	X76, X76.01	
Glaucoma	Glaucoma / raised ocular pressure	1.4 (1.3-1.5)	F93, F93.01, F93.02, F93.03, F93.04	
Sleep apnea syndrome	Sleep apnea syndrome	1.3 (1.2-1.4)	P0601	
Inflammatory bowel disease	Crohn's disease / Ulcerative colitis	1.3 (1.2-1.4)	D94, D94.01, D94.02	
Epilepsy	Epilepsy	1.2 (1.1-1.3)	N88	
Underfeeding/vitamin deficiency	Underfeeding/vitamin deficiency	1.1 (1.0-1.2)	T91, T05	Recode OR connection to episode 12 months after first ICPC
Venous insufficiency	Venous insufficiency<br>Varicose ulcer	1.1 (1.0-1.2)	K99.04 (only K99 not included)<br>S97.01, (Only S97 included, S97.02 Not included)	 Recode OR connection to episode 3 months after first ICPC code
Other psychoactive substance misuse	Substance misuse	1.0 (0.9-1.1)	P19, P19.01, P19.02	
Learning disability' / Mental retardation	Mental retardation<br>Specified learning problems	1.0 (0.9-1.1)	P85<br>P24. P24.01, P24.02, P24.03	
Gout	Gout	0.9 (0.8-1.0)	T92	Recode OR connection to episode ≥3 times / year in 2011, 2012 or 2013. Year starts from First ICPC code. Period between new episodes should be at least 22 days.
Blood(forming organs) and lymphatics disorder	Benign non-specified neoplasm blood/lymph disorder<br>Haemophilia<br>Congenital blood/lymph disorder<br>Purpura/clotting disorder/divergent thrombocytes/ITP	0.8 (0.7-0.9)	B75<br>B83.01<br>B79<br>B83, B83.02, B83.06	Recode OR connection to episode 12 months after first ICPC

Appendix 1 Prevalence estimates with 95% confidence-interval of 76 chronic comorbid diseases, sorted by prevalence (%), continued

Other chronic pulmonary disease	Pulmonary tuberculosis	0.8 (0.7-0.9)	R70	
	Pneumoconiosis		R99.06 (Only R99 not included)	
	Sarcoidosis		R83.02, (Only R83 included)	
Schizophrenia / non-organic psychosis / bipolar disorder	Schizophrenia	0.8 (0.7-0.9)	P72	
	Psychosis non-specified		P98	
	Bipolar disorder		P73.02	
Chronic liver disease	Cirrhosis/steatosis	0.7 (0.6-0.8)	D97, D97.04, D97.05	
Cancer Colorectal	Colon cancer	0.7 (0.6-0.8)	D75	
	Rectal cancer		D75	
Genitourinary cancer, other	Genitourinary cancer, other	0.6 (0.5-0.7)	U75, U77, X77, Y78, Y78.01, Y78.03	
Prostate cancer	Prostate cancer	0.5 (0.4-0.6)	Y77	
Viral hepatitis	Hepatitis B	0.5 (0.4-0.6)	D72.02, D72.04	
	Hepatitis C		D72.03, D72.05	
	Hepatitis		D72 (D72 included D72.01 not included)	
Anorexia or bulimia	Anorexia nervosa	0.5 (0.4-0.6)	T06, T06.01, T06.02	
Carcinoma, other	Carcinoma, other	0.5 (0.4-0.6)	D77.04, T71, W72, L71, L71.01	
Pulmonary cancer	lung / bronchial cancer	0.4 (0.3-0.5)	R84	
Dementia	Alzheimer's disease / Senile dementia/Alzheimer/ Multi-infarct dementia	0.4 (0.3-0.5)	P70.01, P70, P70.02	
Congenital cardiovascular anomaly	Congenital cardiovascular anomaly	0.3 (0.2-0.4)	K73, K73.01, K73.02	
Uterine cervical cancer	Uterine cervical cancer	0.3 (0.2-0.4)	X75	
Bladder cancer	Bladder cancer	0.3 (0.2-0.4)	U76	
Parkinson’s disease	Parkinson's disease	0.2 (0.2-0.2)	N87.01, N87	
Cancer oropharynx, oesophageal, stomach	Cancer of the mouth / pharynx	0.2 (0.2-0.2)	D77.02, D77.03	
	Oesophageal cancer		D77.01, D77	
	Cancer of stomach		D74	
Glomerulonephritis/nephrosis	Glomerulonephritis	0.2 (0.2-0.2)	U88	
Hodgkin disease	Hodgkin disease	0.2 (0.2-0.2)	B72, B72.01, B72.02	
Multiple sclerosis	MS (multiple sclerosis)	0.2 (0.2-0.2)	N86	
Celiac disease	Celiac disease	0.2 (0.2-0.2)	D99.06 (only D99 not included)	
Leukaemia	Leukaemia	0.1 (0.1-0.1)	B73	
Endometrial cancer	Endometrial cancer	0.1 (0.1-0.1)	X77.01	
Ovarian cancer	Ovarian cancer	0.1 (0.1-0.1)	X77.02	
Laryngeal / throat cancer	Laryngeal / throat cancer	0.1 (0.1-0.1)	R85	
Lymphoma / multiple myeloma /other blood cancer	Lymphoma / multiple myeloma /other blood cancer	0.1 (0.1-0.1)	B74.01, B74	
Brain cancer	Brain cancer	0.1 (0.1-0.1)	N74	
Testis Cancer	Testis Cancer	0.1 (0.1-0.1)	Y78.02	
HIV/AIDS	HIV; AIDS	0.1 (0.1-0.1)	B90, B90.01, B90.02	
Metastases; unknown origin	Metastases; unknown origin	0.0 (0.0-0.0)	A79	
Pancreatic cancer	Pancreatic cancer	0.0 (0.0-0.0)	D76	
Anaemia*	Pernicious/folic acid anaemia	-	B81, B81.01, B81.02	Recode OR connection to episode 12 months after first ICPC
	Haemolytic anaemia		B78, B78.01, B78.02, B78.03	

*Data of prevalence of anaemia wasn’t available in the dataset.

Appendix 2: List of disease categories

Disease category	Diseases	Disease category	Diseases
**Cardiovascular**	Hypertension	**Endocrine, metabolic and feeding**	Underfeeding/vitamin deficiency
	Coronary heart disease		Diabetes
	Congenital cardiovascular anomaly		Dyslipidemia
	Heart failure		Obesity
	Stroke&transient ischaemic attack		Thyroid disorder
	Atrial fibrillation	**Neurological**	Dementia
	Heart valve disease		Epilepsy
	Venous insufficiency		Migraine
	Peripheral vascular disease		Parkinson's disease
**Respiratory**	COPD		Multiple sclerosis
		**Blood(forming organs) and Lymphatics**	Anaemia
	Sleep apnea syndrome		Blood(forming organs) and lymphatics disorder
	Chronic sinusitis	**Infectious**	Viral hepatitis
	Other chronic pulmonary disease		HIV/AIDS
	Bronchiectasis / chronic bronchitis	**Non-pulmonary cancer**	Testis Cancer
**Mental Health**	Depression		Cancer oropharynx, oesophageal, stomach
	Anxiety		Cancer Colorectal
	Alcohol problems		Pancreatic cancer
	Other psychoactive substance misuse		Laryngeal / throat cancer
	Schizophrenia / non-organic psychosis / bipolar disorder		Breast cancer
	Anorexia or bulimia		Ovarian cancer
	Personality disorder		Endometrial cancer
	Learning disability' / Mental retardation		Uterine cervical cancer
**Musculoskeletal**	Rheumatoid arthritis, other inflammatory polyarthritis & systemic connective tissue disorders		Prostate cancer
	Gout		Bladder cancer
	Osteoporosis/osteopenia		Genitourinary cancer, other
	Osteoarthritis		Brain cancer
**Eye and Ear**	Hearing loss		Hodgkin disease
	Glaucoma		Leukaemia
	Blindness & low vision		Lymphoma / multiple myeloma /other blood cancer
**Urogenital (Male and female)**	Chronic kidney disease		Metastases; unknown origin
	Glomerulonephritis/nephrosis		Carcinoma, other
	Recurrent urinary tract infection		Skin cancer
	Prostate disorders	**Pulmonary cancer**	Pulmonary cancer
**Skin**	Eczema		
	Psoriasis		
	Other chronic skin disease / neoplasm (sub)cutis		

Appendix 3 Prevalence estimates of 76 comorbid diseases, sorted by total prevalence (%). Odds ratios for female sex were adjusted for age. Odds ratios marked **bold** were statistically significant. This appendix is an extension of table 3.

Comorbidity	Total prevalence, (%)	Prevalence in male, (%)	Prevalence in female, (%)	Odds ratio female sex (95%-CI)
Hypertension	20.1	18.0	21.6	**1.26 (1.18-1.34)**
Osteoarthritis	11.5	8.4	13.7	**1.80 (1.66-1.95)**
Eczema	11.5	10.7	12.1	**1.17 (1.09-1.25)**
Dyspepsia, gastroesophageal reflux	10.7	10.1	11.2	**1.10 (1.02-1.18)**
COPD	9.5	10.7	8.6	**0.72 (0.67-0.78)**
Diabetes	8.5	8.6	8.4	0.92 (0.85-1.00)
Dyslipidemia	6.9	6.9	6.8	0.93 (0.85-1.02)
Chronic sinusitis	6.6	4.7	7.9	**1.73 (1.57-1.90)**
Obesity	6.5	4.4	8.0	**1.88 (1.70-2.07)**
Coronary heart disease	6.5	8.3	5.3	**0.52 (0.48-0.58)**
Blindness & low vision	6.1	5.6	6.4	1.05 (0.94-1.16)
Peripheral vascular disease	5.5	5.5	5.6	0.96 (0.87-1.06)
Irritable bowel syndrome	5.4	2.7	7.3	**2.84 (2.53-3.20)**
Thyroid disorder	5.1	1.6	7.4	**5.00 (4.32-5.80)**
Anxiety	4.9	3.2	6.0	**1.96 (1.75-2.19)**
Depression	4.6	2.9	5.7	**2.05 (1.83-2.31)**
Chronic kidney disease	4.4	3.6	4.9	**1.30 (1.16-1.47)**
Hearing loss	4.0	4.6	3.6	**0.67 (0.60-0.75)**
Stroke&transient ischaemic attack	3.4	3.5	3.3	**0.87 (0.77-0.99)**
Psoriasis	3.4	3.4	3.3	0.95 (0.84-1.08)
Skin cancer	3.0	2.9	3.0	0.98 (0.86-1.12)
Osteoporosis/osteopenia	2.9	1.0	4.2	**4.14 (3.44-4.98)**
Atrial fibrillation	2.9	3.3	2.6	**0.68 (0.59-0.78)**
Rheumatoid arthritis, other inflammatory polyarthritis & systemic connective tissue disorders	2.2	1.6	2.6	**1.64 (1.40-1.93)**
Diverticular disease of intestine	2.2	1.9	2.3	1.15 (0.98-1.34)
Migraine	2.1	0.7	3.1	**4.39 (3.54-5.44)**
Prostate disorders	2.0	4.9	-	-
Personality disorder	1.9	1.6	2.1	**1.40 (1.18-1.65)**
Heart failure	1.8	1.8	1.8	0.85 (0.72-1.02)
Recurrent urinary tract infection	1.8	0.3	2.8	**9.13 (6.64-12.55)**
Other chronic skin disease / neoplasm	1.7	1.6	1.8	1.16 (0.97-1.37)
Heart valve disease	1.7	1.8	1.7	0.86 (0.72-1.02)
Alcohol problems	1.7	2.6	1.0	**0.38 (0.32-0.45)**
Bronchiectasis / chronic bronchitis	1.6	1.5	1.6	1.08 (0.90-1.29)
Breast cancer	1.6	0.0	2.6	-
Glaucoma	1.4	1.4	1.4	0.91 (0.75-1.10)
Sleep apnea syndrome	1.3	2.1	0.8	**0.39 (0.32-0.48)**

Inflammatory bowel disease	1.3	1.1	1.5	**1.34 (1.10-1.64)**
Epilepsy	1.2	1.3	1.1	0.88 (0.72-1.08)
Underfeeding/vitamin deficiency	1.1	0.5	1.5	**3.30 (2.50-4.34)**
Venous insufficiency	1.1	0.7	1.3	**1.63 (1.29-2.06)**
Other psychoactive substance misuse	1.0	1.6	0.6	**0.35 (0.28-0.44)**
Learning disability / Mental retardation	1.0	1.2	0.9	**0.78 (0.63-0.97)**
Gout	0.9	1.6	0.4	**0.22 (0.17-0.29)**
Blood(forming organs) and lymphatics	0.8	0.5	1.0	**1.83 (1.39-2.42)**
Other chronic pulmonary disease	0.8	0.9	0.7	0.79 (0.62-1.02)
Schizophrenia / non-organic psychosis	0.8	0.8	0.7	0.87 (0.67-1.11)
Chronic liver disease	0.7	0.9	0.6	**0.67 (0.52-0.87)**
Colorectal cancer	0.7	0.8	0.6	**0.75 (0.58-0.98)**
Genitourinary cancer, other	0.6	0.3	0.7	**2.03 (1.46-2.84)**
Prostate cancer	0.5	1.2	-	-
Viral hepatitis	0.5	0.5	0.5	0.93 (0.68-1.27)
Anorexia or bulimia nervosa	0.5	0.0	0.8	**27.27 (10.10-73.63)**
Carcinoma, other	0.5	0.4	0.5	1.41 (1.01-1.98)
Pulmonary cancer	0.4	0.5	0.3	**0.59 (0.42-0.84)**
Dementia	0.4	0.4	0.5	0.99 (0.70-1.40)
Congenital cardiovascular anomaly	0.3	0.4	0.3	0.81 (0.55-1.19)
Uterine cervical cancer	0.3	-	0.5	-
Bladder cancer	0.3	0.4	0.2	**0.39 (0.25-0.59)**
Parkinson's disease	0.2	0.3	0.2	**0.55 (0.36-0.86)**
Cancer oropharynx, oesophageal, stomach	0.2	0.2	0.2	0.88 (0.56-1.38)
Glomerulonephritis/nephrosis	0.2	0.3	0.2	0.69 (0.44-1.09)
Hodgkin disease	0.2	0.3	0.2	0.63 (0.40-1.00)
Multiple sclerosis	0.2	0.1	0.3	**4.28 (2.03-9.04)**
Coeliac disease	0.2	0.1	0.2	1.22 (0.71-2.11)
Leukaemia	0.1	0.2	0.1	**0.49 (0.27-0.88)**
Endometrial cancer	0.1	-	0.2	-
Ovarian cancer	0.1	-	0.1	-
Laryngeal / throat cancer	0.1	0.1	0.0	**0.32 (0.14-0.76)**
Lymphoma/multiple myeloma/other blood cancer	0.1	0.1	0.1	0.78 (0.34-1.81)
Brain cancer	0.1	0.1	0.1	0.91 (0.38-2.16)
Testis Cancer	0.1	0.1	-	-
HIV/AIDS	0.1	0.1	0.0	**0.35 (0.13-0.92)**
Metastases; unknown origin	0.0	0.0	0.1	2.63 (0.74-9.31)
Pancreatic cancer	0.0	0.0	0.1	1.64 (0.51-5.23)
Anaemia*	-	-	-	-

*Data of prevalence of anaemia wasn't available in the dataset.
